# Supplementary material for: An Investigation into the Influence of Different Types of Nesting Materials upon the Welfare of Captive Chimpanzees (Pan troglodytes)
Source: Animals (Basel). 2021 Jun 20;11(6):1835. doi: 10.3390/ani11061835 (PMC8235373; doi:10.3390/ani11061835)
Supplement: Supplementary file 1 [file animals-11-01835-s001.zip › animals-1144988-supplementary.pdf]

Table S1. Ethogram used during the data collection sessions on western chimpanzees (*Pan troglodytes verus*), between November 2019 and March 2020, at Tacugama Chimpanzee Sanctuary (TCS), Sierra Leone.

**Behavioural Category**	**Subcategory**	**Behaviour**[1]	**Definition**
Individual *Not interacting with others*		Feed (+)	To eat or drink
		Forage (+)	Searching for food
		Vigilant	Individual is in a heightened state of alert, watching his/her surroundings attentively
		Locomotion (+)	Movement from a point A to a point B, by means of walking, running, swinging, or climbing
		Solitary play (+)	Play behaviour without the involvement of another individual
		Manipulate Object (+)	To explore and hold an object with hands and/or feet
	Self-Directed	Autogrooming (-)	To groom oneself
		Scratch (-)	To scratch oneself
		Genital manipulation (-)	Manipulation of own genitals - includes masturbation
		Wadging (-)	Re-chewing food into a ball
Abnormal		Stereotyped movement (-)	Repetition of an unnecessary body motion numerous times e.g. rocking, pacing
		Urophagy/ Coprophagy (-)	Ingestion of urine (urophagy) or faeces (coprophagy), belonging to one's self or another
		Regurgitation (-)	Expulsion of previously digested food to an exterior substrate (e.g. floor, platform), and its subsequent consumption
		Abnormal Genital Manipulation (-)	Manipulation of own genitals in an abnormal manner e.g. Masturbating with faeces
		Abnormal Object Manipulation (-)	Manipulation of own, or another individual's, faeces, or urine
Social *Interacting with others*	Affiliative	Allogrooming	To groom another individual
		Play Attempt	Game incitement behaviour towards another individual
		Play	Playful interactions, often accompanied by a play face, including chasing, wrestling, tickling
	Sexual	Copulation/ Attempted Copulation	Sexual intercourse
		Genital Inspection	Examination of another individual's genitals
		Genital Presentation	Directing own genitals towards another individual
	Agonistic	Hit	To hit or slap another individual
		Kick	To kick another individual
		Bite	To bite another individual
		Chase	To pursue another individual
		Flee	To escape from an individual
		Display	Intimidatory behaviour, including swaying, slapping substrate, shaking enclosure bars, often accompanied by piloerection
		Fight	Repeated hits or kicks towards another individual
		Scream	Screaming vocalization associated with anxiousness - often performed after fighting

Table S1 (cont). Ethogram used during the data collection sessions on western chimpanzees (*Pan troglodytes verus*), between November 2019 and March 2020, at TCS, Sierra Leone.

Enrichment	**Cover (+)**	**To cover body with enrichment**
	Make nest (+)	To make nest using the enrichment
	Eat / Chew (+)	To chew, or wadge on enrichment material
	Tear with mouth (+)	Individual alters initial size and structure of material using their mouth
	Tear with hands (+)	Individual alters initial size and structure of material with the use of hands
	Manipulate with hands (+)	Any other manipulation/ exploration of enrichment with hands, excluding tearing
	Travel (+)	To purposefully move enrichment from one location to another
Rest	Sit (-)	When not actively engaging in any other behaviour, apart from sitting
	Stand (-)	When not actively engaging in any other behaviour, apart from standing
	Lie (-)	When not actively engaging in any other behaviour, apart from lying - includes sleeping

[1] (+) refers to positive individual behaviours, and (-) refers to negative individual behaviours
